# Differential Neural Correlates Underlie Judgment of Learning and Subsequent Memory Performance

**DOI:** 10.3389/fpsyg.2015.01699

**Published:** 2015-11-09

**Authors:** Haiyan Yang, Ying Cai, Qi Liu, Xiao Zhao, Qiang Wang, Chuansheng Chen, Gui Xue

**Affiliations:** ^1^State Key Laboratory of Cognitive Neuroscience and Learning and IDG/McGovern Institute for Brain Research, Beijing Normal UniversityBeijing, China; ^2^Center for Collaboration and Innovation in Brain and Learning Sciences, Beijing Normal UniversityBeijing, China; ^3^Department of Psychology and Social Behavior, University of California, Irvine, IrvineCA, USA

**Keywords:** judgment of learning, fMRI, subsequent memory effect, default-model network, processing fluency

## Abstract

Judgment of learning (JOL) plays a pivotal role in self-regulated learning. Although the JOLs are in general accurate, important deviations from memory performance are often reported, especially when the JOLs are made immediately after learning. Nevertheless, existing studies have not clearly dissociated the neural processes underlying subjective JOL and objective memory. In the present study, participants were asked to study a list of words that would be tested 1 day later. Immediately after learning an item, participants predicted how likely they would remember that item. Critically, the JOL was performed on only half of the studied items to avoid its contamination on subsequent memory. We found that during encoding, compared to items later judged as “will be forgotten,” those judged as “will be remembered” showed stronger activities in the default-mode network, including the ventromedial prefrontal cortex (PFC) and posterior cingulate cortex, as well as weaker functional connectivity between the left dorsolateral PFC and the visual cortex. The exact opposite pattern was found when comparing items that were actually remembered with those that were later forgotten. These important neural dissociations between JOL and memory performance shed light on the neural mechanisms of human metamemory bias.

## Introduction

Self-knowledge is an integral part of human cognition. For example, to become a sophisticated and effective learner, one needs to be able to accurately assess or monitor the state of one’s learning, which serves as the basis for selecting more effective learning strategies, and allocating proper cognitive and neural resources to the learning materials (Nelson, 1990; Bjork et al., 2013). A commonly used measure of memory monitoring is the judgment of learning (JOL), which is participants’ assessment of the likelihood of remembering a specific item on a future test (Nelson, 1990). Many studies have shown that JOLs are both moderately accurate in predicting future memory performance and generally sensitive to the manipulations that affect actual learning and memory performance (Nelson and Dunlosky, 1991; Dunlosky and Nelson, 1994; Koriat, 1997). It has been also shown that JOL capacity is positively correlated with individuals’ memory performance (Thiede et al., 2003).

Nevertheless, important dissociations have been observed between predicted performance (JOLs) and actual performance (Carroll et al., 1997; Benjamin et al., 1998; Simon and Bjork, 2001; Kornell et al., 2011; Besken and Mulligan, 2013; Carpenter et al., 2013; Castel et al., 2013; Mueller et al., 2014). One important factor that affects the JOL accuracy is when the JOLs are made. It has been found that compared to JOLs that were made right after learning (i.e., immediate JOLs), delayed JOLs that were made 30 s after learning were significantly more accurate (Nelson and Dunlosky, 1991), and more sensitive to the manipulations that could affect memory performance (Dunlosky and Nelson, 1994). One explanation is that JOLs made immediately after study are based on information in working memory that affects JOLs but is not predictive of eventual memory performance. However, when the JOLs are delayed, they are based on information retrieved from long-term memory that might more accurately assess the effects of the study activities on subsequent retention (Dunlosky and Nelson, 1994).

These studies suggest that differential cognitive and neural processes underlying JOL, in particular, immediate JOL and memory encoding. Consistently, cognitive studies have suggested that JOLs are often based on ease of processing (i.e., “processing fluency heuristic”) (Kelley and Jacoby, 1996; Hertzog et al., 2003; Rhodes and Castel, 2008; Kornell et al., 2011; Sungkhasettee et al., 2011; Besken and Mulligan, 2013), whereas effective learning needs desirable difficulties (Bjork, 1994; McDaniel and Butler, 2011). For example, when related word pairs were compared with unrelated word pairs (Dunlosky and Matvey, 2001), common concrete words with rare abstract words (Begg et al., 1989), massed repetition with spaced repetition (Simon and Bjork, 2001), the former learning material or learning method was judged as easier but in fact showed worse performance than the latter.

At the neural level, evidence from psychopharmacological (Dunlosky et al., 1998; Izaute and Bacon, 2004; Mintzer and Griffiths, 2005; Mintzer et al., 2010) and neuropsychological studies (Vilkki et al., 1999; Pannu and Kaszniak, 2005; Andrés et al., 2010; Howard et al., 2010) indicates that the memory and metamemory processes depend upon at least partially distinct neural circuitries (Chua et al., 2014). Both nitrous oxide and benzodiazepine lorazepam impair memory performance, but not the ability to form accurate JOLs (Dunlosky et al., 1998; Izaute and Bacon, 2004; Mintzer and Griffiths, 2005). Individuals with frontal lobe lesions are impaired in JOLs, despite normal recognition-memory performance, whereas patients with posterior or temporal lobe lesions have impaired memory but intact JOLs (Vilkki et al., 1999; Andrés et al., 2010).

Only a few functional imaging studies have been conducted to specify the neural circuitry of JOL, all using the immediate JOL paradigm (Kao et al., 2005; Skavhaug et al., 2010, 2013; Do Lam et al., 2012). In the first neuroimaging study on the neural basis of JOL (Kao et al., 2005), participants were asked to estimate during encoding whether they would later be able to recognize each presented item. Brain activation in the ventromedial prefrontal cortex (PFC) increased with predicted memory success during encoding, whereas actual subsequent memory was associated with enhanced activity in the medial temporal lobes (MTL). With a similar paradigm and high temporal resolution EEG, another study revealed temporal dissociations in processes that predicted later JOL rating (“will remember” vs. “will forget”) and that predicted later actual memory (Skavhaug et al., 2010). One limitation of these studies was that JOL was performed at the time of stimulus encoding, which did not allow for a clear separation of the neural correlates of encoding and JOL.

In a recent study, JOLs were separated from encoding trials by a short temporal delay (Do Lam et al., 2012), and the result showed that later actual memory success was associated with increased hippocampal activation, but predicted memory success was accompanied by increased activation in mPFC, orbital frontal cortex, and anterior cingulate cortex. Although this design overcame the weakness of the previous two studies (i.e., inability to separate JOL from encoding), the JOL would involve a retrieval process (Spellman and Bjork, 1992; Benjamin et al., 1998). This could serve as an additional encoding event, which could enhance subsequent memory, affect JOL accuracy, and contaminate the estimation of the neural mechanisms underlying memory performance.

The present study was designed to overcome both weaknesses in the previous studies. Subjects were asked to study a list of words. Only half of the words were followed by an immediate JOL. One day after learning, memory for all materials was tested. With this design, we could clearly separate the encoding and JOL processes and also avoid the contamination of the JOL processes on memory encoding. We predicted that the neural processes supporting later JOL would be dissociated from those supporting memory encoding. In particular, if the processing fluency heuristic hypothesis is true, we should predict that strong encoding-related processes would lead to better actual memory but lower JOL. In addition, by comparing the brain regions involved in memory encoding with those involved in JOL, we could further elucidate the neural substrates underlying JOL and memory encoding.

## Materials and Methods

### Participants

Twenty-six college students (12 males, mean age = 21.6 ± 1.8 years, ranging from 18 to 25 years) were recruited for this study. All participants had normal or corrected-to-normal vision, and were self-reported to be right-handed and to have no previous history of neurological or psychiatric diseases. One additional subject was recruited but his data were discarded due to excessive head motion. Informed written consent was obtained before the experiment. This study was approved by the Institutional Review Board of the State Key Laboratory of Cognitive Neuroscience and Learning at Beijing Normal University.

### Materials

A total of 360 two-character Chinese nouns were used as learning material. Half of the words were used for the learning condition (LRN), and the other half were used for the learning and JOL condition (LRN + JOL). They were matched on frequency and abstractness and counterbalanced across subjects. All words were presented visually in white color on a black background. Another 360 words were used as foils in the recognition memory test. To minimize the primacy and recency effects, three words were added at the beginning and the end of each encoding run respectively, and these words were excluded from both behavioral and MRI analyses.

### fMRI Procedures

**FIGURE 1:**
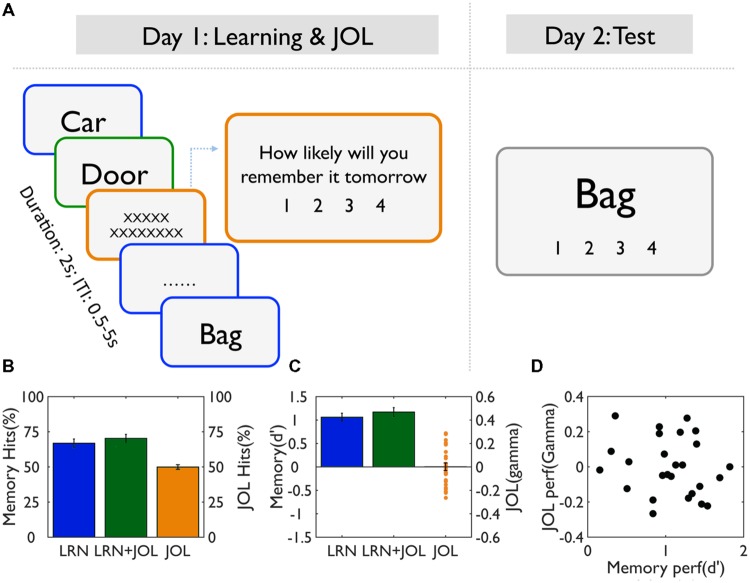
**Task procedure and behavioral results. (A)** Experimental design. On Day 1, participants studied a list of words for a later memory test, using a semantic judgment task (concrete vs. abstract). For half of the trials (LRN + JOL), participants were asked to make JOL right after learning, that is, to predict how likely they would remember the items tomorrow. No JOL was performed on the other half trials (LRN) to avoid the contamination of JOL on memory performance. On Day 2, participants were asked to perform the recognition memory test on all studied trials. **(B)** Memory performance for the LRN and LRN + JOL trials as measured by hits rate, and JOL accuracy as measured by JOL hits (i.e., correctly predicted items). **(C)** Memory accuracy for the LRN and LRN+JOL trials as measured by d’, and JOL accuracy as measured by gamma. **(D)** The scatter plot of the relationship between memory accuracy (d’) and JOL accuracy (gamma).

Participants lay supine on the scanner bed, and viewed visual stimuli back-projected onto a screen through a mirror attached onto the head coil. Foam pads were used to minimize head motion. Stimulus presentation and timing were achieved using Matlab (Mathworks) and Psychtoolbox^1^ on an IBM-compatible PC. During the scan, participants were instructed to judge whether each word represented a concrete or abstract concept, by pressing a button with their index fingers (**Figure 1**). They were also explicitly told that a recognition memory test would be conducted 24 h later to test their memory. For half of the words, after the learning event, subjects were asked to perform the JOL, that is, to predict how likely they would remember each of the items tomorrow on a four-point scale with 1 = “will be absolutely forgotten” and 4 = “will be absolutely remembered”. Participants’ responses were collected online using an MRI-compatible button box. The hands used to indicate an abstract or concrete response as well as low vs. high JOL were counterbalanced across participants.

Event-related design was used in this study. For each trial, the stimulus was presented up to 2 s until a valid response was received, which was then followed by a cross fixation at the center of the screen until the designated onset time of the next stimulus. For each JOL trial, subjects had up to 2 s to make the judgment. Random jitters from 0.5 to 6.5 sec (mean: 2 s) were added between trials and the sequence was optimized for design efficiency (Dale, 1999) using an in-house program. This would allow us to effectively separate the learning and JOL events. In total, participants finished three runs of the learning and JOL tasks, each with 126 learning trials (including the 3 fillers at the beginning and 3 at the end of the sequence) and 60 JOL trials. Each run lasted around 12.5 min.

### MRI Acquisition

Imaging data were acquired on a 3.0 T Siemens MRI scanner in the MRI Center at Beijing Normal University. A single-shot T2-weighted gradient-echo, EPI sequence was used for functional imaging acquisition with the following parameters: TR/TE/θ = 2000 ms/25 ms/90°, FOV = 192 × 192 mm, matrix = 64 × 64, and slice thickness = 3 mm. Forty-one contiguous axial slices parallel to the AC-PC line were obtained to cover the whole cerebrum and partial cerebellum. Anatomical MRI was acquired using a T1- weighted, three-dimensional, gradient-echo pulse-sequence (MPRAGE). The parameters for this sequence were: TR/TE/θ = 2530ms/3.09ms/10°, FOV = 256 × 256 mm, matrix = 256 × 256, and slice thickness = 1 mm. In total, 208 sagittal slices were acquired to provide high-resolution structural images of the whole brain.

### Post-scan Memory Tests

Twenty-four hours after the encoding task, participants were called back to the lab to complete the recognition test. All studied words and an equal number of foils were randomly mixed together. Participants were asked to decide whether the word presented on the screen was studied or not on a four-point scale, with 1 = “definitely new,” and 4 = “definitely old.” The stimulus remained on the screen until a response was made or 10 s had lapsed. The next item appeared after a 1 s delay.

### Behavioral Data Analysis

Two indices were used to describe memory performance. The first index was the proportion of correct hits (scored 3 and 4). Because this result may be biased by individuals’ response criteria (Snodgrass and Corwin, 1988), another unbiased discriminability index (d’) was computed using the following formula: d’ = Z(hit rate) – Z(false alarm rate).

There were also two indices of JOL. The first index was the JOL accuracy, which was the proportion of trials in which JOL matched the actual memory performance. Following the existing literature (Kao et al., 2005), we also calculated the Goodman–Kruskal gamma for each participant, using the following equation: G = (C - D)/(C + D), where C is the number of concordant pairs and D is the number of discordant pairs. The gamma statistic indicates the strength of association between two ordinal variables and is the preferred measure in metamemory research (Nelson, 1984). A higher gamma value thus suggests that the JOL score matches the actual memory performance more closely.

### fMRI Data Preprocessing and Statistical Analysis

Image preprocessing and statistical analyses were carried out using FEAT (FMRI Expert Analysis Tool) version 5.98, part of the FSL (FMRIB software library, version 4.1).^2^ The first three volumes before the task were automatically discarded by the scanner to allow for T1 equilibrium. The remaining images were then realigned to correct for head movements (Jenkinson and Smith, 2001). Translational movement parameters never exceeded 1 voxel in any direction for any participant or session. Data were spatially smoothed using a 5-mm full-width-half-maximum (FWHM) Gaussian kernel. The spatially smoothed data were then filtered temporally using a non-linear high-pass filter with a 60-s cut-off. A two-step registration procedure was used whereby EPI images were first registered to the MPRAGE structural image, and then into the standard MNI space, using affine transformations (Jenkinson and Smith, 2001). Registration from structural images to the standard space was further refined using FNIRT non-linear registration (Andersson et al., 2007a,b). Statistical analyses were performed in the native image space, with the statistical maps normalized to the standard space prior to higher-level analyses.

The general linear model within the FILM module of FSL was used to model the data. For all analyses, events were modeled at the time of the stimulus onset and convolved with canonical hemodynamic response function (double gamma function). Temporal derivatives were included as covariates of no interest to improve statistical sensitivity. The six filler words were modeled as a single nuisance variable. Null events were not explicitly modeled and thus served as a baseline.

The first analysis was to examine the subsequent memory effect and the subsequent JOL effect. At the encoding stage, to examine the subsequent memory effect, we separated the LRN trials into remembered trials (R, scored ≥3 in the recognition memory test) and forgotten trials (F, scored ≤2). To examine the subsequent JOL effect, we separated the LRN + JOL trials into “will be remembered” trials (r, scored ≥3 in JOL) and ”will be forgotten” trials (f, scored ≤2). Three contrasts were defined, including the subsequent memory effect (1 -1 0 0, for R, F, r, and f, respectively), the subsequent JOL effect (0 0 1 -1), and their direct comparison (1 -1 -1 1). At the JOL stage, we separated the trials into four types based on the combination of memory performance (R vs. F) and JOL (r vs. f), including Rr, Rf, Fr, and Ff. We defined three contrasts, including the high vs. low JOL (1 -1 1 -1, for Rr, Rf, Fr, and Ff, respectively), correct vs. incorrect JOL (1 -1 -1 1), and remembered vs. forgotten memory (1 1 -1 -1).

In an additional analysis, we examined whether JOL could affect the subsequent memory effect. This was achieved by comparing the subsequent memory effect based on the LRN trials and that based on the LRN + JOL trials. The GLM model was similar to the above model except that in this analysis, the LRN + JOL trials were grouped based on the actual memory performance (remembered vs. forgotten), not the JOL. Three contrasts were defined, including the subsequent memory effect based on LRN trials (1 -1 0 0), the subsequent memory effect based on LRN + JOL trials (0 0 1 -1), and their direct comparison (1 -1 -1 1).

Using a fixed-effects model, cross-run averages for a set of contrast images were created for each participant. These contrast images were then entered into a random-effects model for group analysis, using FMRIB’s Local Analysis of Mixed Effects (FLAME) estimation. Group images were thresholded using cluster detection statistics, with a height threshold of *Z* > 2.3 and a cluster probability of *p* < 0.05, corrected for whole-brain multiple comparisons using Gaussian Random Field Theory (GRFT).

### Psychophysiological Interaction (PPI) Analysis

Conjunction analysis revealed that one cluster in the left dorsolateral PFC (DLPFC) showed both the subsequent memory effect and the subsequent JOL effect. We then examined the functional connectivity with this region using psychophysiological interactions (PPI) analysis (Friston et al., 1997). The left DLPFC cluster from the conjunction analysis [MNI (center of gravity): -38, 12, 32) was defined as the seed region. The time course of DLPFC activity was defined as the physiological variable and its interaction with memory performance (R, F) and JOL (r, f) was defined as the PPI variable, using a generalized form of context-dependent PPIs (McLaren et al., 2012).

### Region of Interests (ROI) Analysis

Group analyses revealed several regions showing differences in activation level or connectivity strength between the subsequent memory effect and the subsequent JOL effect. Their response profiles were further probed in ROI analyses. ROI analyses were performed by extracting parameter estimates (betas) of each event type from the fitted model and averaging across all voxels in each cluster for each participant. Percent signal changes were calculated using the following formula: [contrast image/(mean of run)] × ppheight × 100%, where ppheight is the peak height of the hemodynamic response versus the baseline level of activity (Mumford, 2007).

## Results

### Behavioral Results: Memory Performance and JOL Accuracy

On average, subjects remembered 66.8% ± 15% of the learning only (LRN) trials, and the d’ was 1.06 ± 0.42, suggesting subjects performed moderately well in the memory task. In contrast, JOL accuracy was 49.9% ± 8%, not significantly different from chance [*t*(25) = 0.04, *p* = 0.97]. Similarly, average gamma was 0.001 ± 0.16, not significantly different from zero [*t*(25) = 0.05, *p* = 0.96; **Figures 1B,C**].

The low JOL accuracy might be due to the fact that participants overall underestimated their memory performance [*R* = 120.26 ± 27.46, but *r* = 82.81 ± 29.02, *t*(25) = 6.41, *p* < 0.0001]. To further examine whether participants’ JOLs were sensitive to subsequent memory performance despite the overall low accuracy, we calculated the JOL ratings for words with different subsequent memory scores (words scored 1 and 2, i.e., forgotten words, were analyzed together as there were very few words scored 1). The only significant result was that subjects gave more low JOL ratings (i.e., 1) to better remembered trials (i.e., scored 4) (**Table 1**). There were no other significant differences in the percentage of each type of ratings given to each memory performance (when the JOL ratings were treated as categorical variables), the mean JOL scores (when the ratings were treated as continuous variables) or the JOL accuracy. These results suggest that subjects overall could not predict subsequent memory performance.

**Table 1:** Judgment of learning (JOL) rating as a function of actual memory performance.

	JOL rating				JOL score	JOL accuracy
Memory	1	2	3	4		
*F* (Scored 1 or 2)	8.72%	42.56%	41.90%	6.82%	2.43	51.28%
*R* (Scored 3)	8.23%	44.50%	40.99%	6.29%	2.42	47.27%
*R* (Scored 4)	11.70%	43.16%	39.67%	5.46%	2.37	45.14%
*F*-value	3.23	0.23	0.32	1.23	1.72	0.68
*P*-value	0.05^∗^	0.80	0.73	0.30	0.19	0.51

*^∗^*P* ≤ 0.05.*

There were sizable individual differences in terms of both memory performance (d’ ranged from 0.15 to 1.82) and JOL accuracy (gamma ranged from -0.26 to 0.29), but they were not correlated with each other across subjects (*r* = -0.18, *p* = 0.37) (**Figure 1D**).

### JOL Processes Enhanced Memory Performance

Compared to the LRN trials, the LRN + JOL trials were more likely to be judged as old trials [*t*(25) = 2.91, *p* = 0.0075], and their mean d’ score was also higher [*t*(25) = 2.55, *p* = 0.017] (**Figures 1B,C**). However, subsequent memory (remembered vs. forgotten) by subjectivity (memory vs. JOL) ANOVA revealed no significant subsequent memory effect, JOL effect, or their interaction in either accuracy or RT during memory encoding (i.e., semantic judgment) (all *p*s > 0.15). These results suggest that the LRN trials and LRN + JOL trials were matched, and that the behavioral indices at the encoding stage could not predict subsequent memory or subsequent JOL.

### fMRI Results: Neural Activities Underlying Subsequent Memory Performance

**FIGURE 2:**
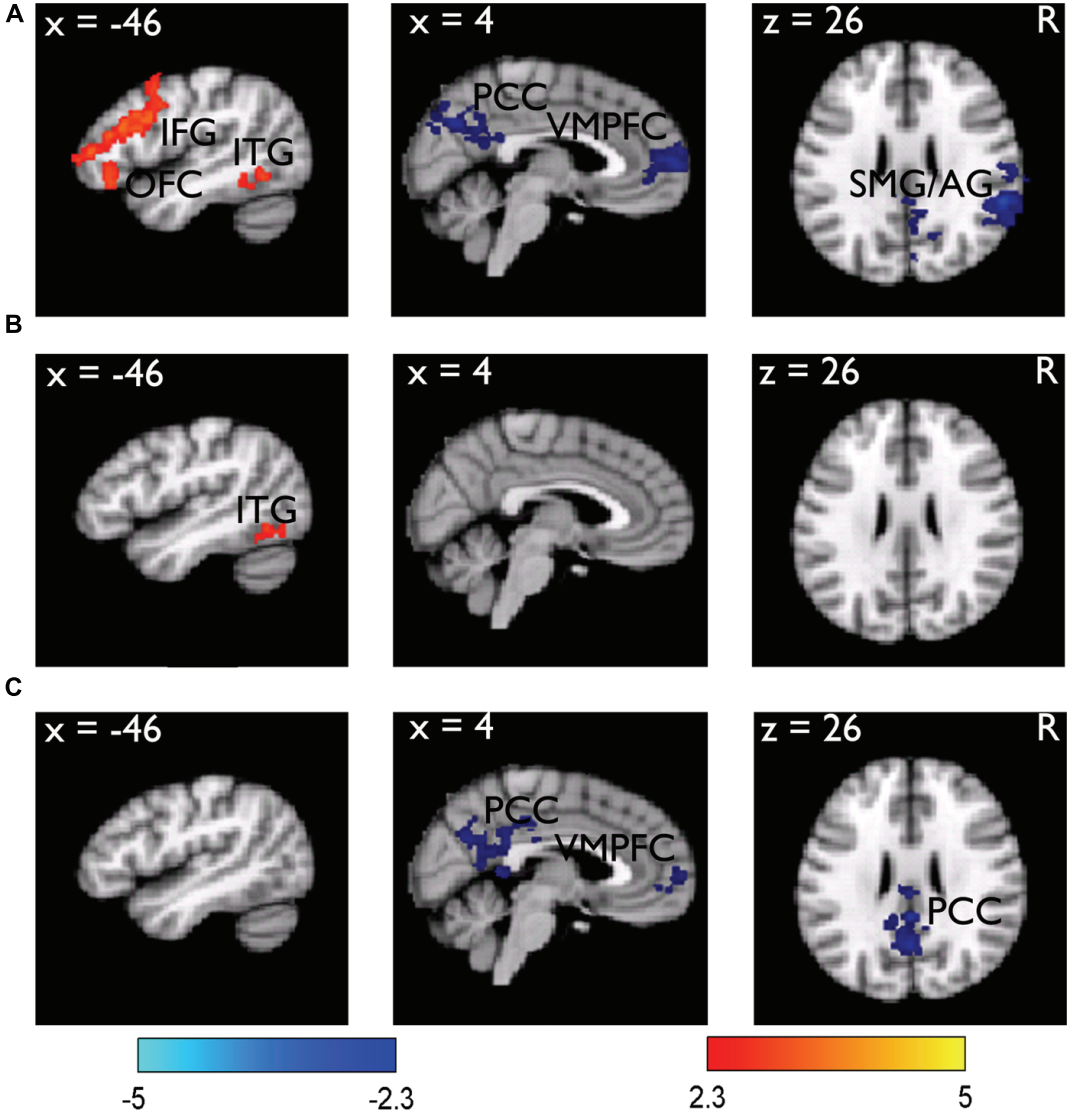
**Brain regions related to the subsequent memory effect. (A)** Brain regions showing stronger (red-yellow color) and weaker (blue color) activity during learning/encoding for subsequently remembered than forgotten LRN items. The results are overlain on the sagittal and axial slices of the group mean structural image. All activations were thresholded at *Z* > 2.3 (whole-brain corrected *p* < 0.05). **(B)** The subsequent memory effect obtained from the LRN + JOL trials, showed an overall reduction. **(C)** Direct comparison of the subsequent memory effect between the LRN and LRN + JOL trials revealed stronger deactivation for the LRN trials.

We then examined the core hypothesis regarding the dissociated neural processes underlying subsequent memory and subsequent JOL. We first examined the subsequent memory effect by comparing the neural activities for subsequently remembered (R) vs. forgotten (F) trials from the LRN trials. Results revealed that items with better subsequent memory performance (i.e., R minus F) elicited stronger activation in the left inferior frontal gyrus (IFG) (MNI: -42, 32, 16, *Z* = 4.13) that extended ventrally to the lateral orbital frontal cortex (LOFC) (MNI: -44, 32, -14, *Z* = 3.46), and the left inferior temporal gyrus (LITG) (MNI: -40, -54, -8, *Z* = 3.54). In contrast, items with worse subsequent memory performance (i.e., F minus R) elicited stronger activation in the right supramarginal gyrus/angular gyrus (SMG/AG) (MNI: 58, -42, 26, *Z* = 4.73), the posterior cingulate cortex (PCC) (MNI: 8, -72, 34, *Z* = 3.76), and the ventromedial prefrontal cortex (VMPFC) (MNI: 2, 62, 6, *Z* = 3.81) (**Figure 2A**).

Consistent with the hypothesis that the JOL could contaminate the subsequent memory effect, we found that the subsequent memory effect based on the LRN + JOL trials was weaker compared to that obtained from the LRN trials. There was no significant activation in the left IFG and no significant deactivation in any of the above regions (**Figure 2B**). Direct comparison between the two subsequent memory effects revealed stronger deactivation in the VMPFC (MNI: 2, 62, 4, *Z* = 3.32), the PCC (MNI: 2, -42, 26, *Z* = 3.59), and the precuneus (MNI: 0, -60, 28, *Z* = 3.67) for the LRN trials than the LRN + JOL trials (**Figure 2C**).

### Neural Activities Underlying the Subsequent JOL Effect

**FIGURE 3:**
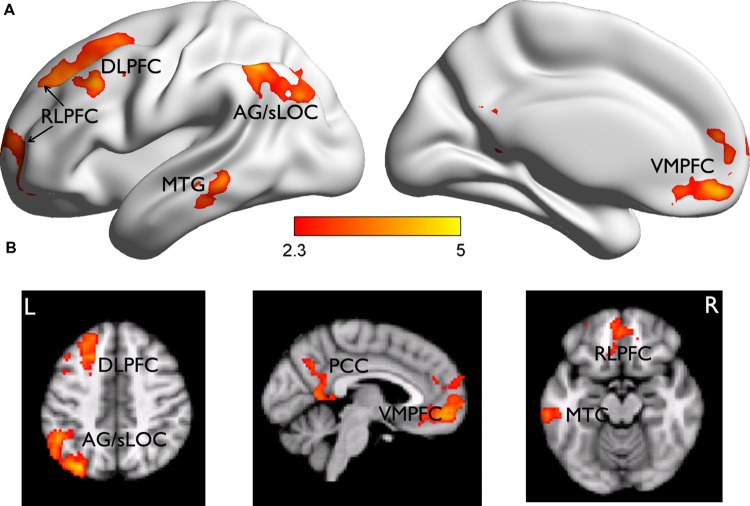
**Brain regions related to the subsequent JOL effect. (A)** Brian regions showing stronger activity for trials judged as “will be remembered” than those judged as “will be forgotten.” They are rendered onto a population-averaged surface atlas (Xia et al., 2013). **(B)** The same results are overlain on the axial and sagittal slices of the group mean structural image. All activations were thresholded at *Z* > 2.3 (whole-brain corrected *p* < 0.05).

The subsequent JOL effect was examined by comparing the neural activities for trials subsequently judged as “will be remembered” (r) vs. those as “will be forgotten” (f) from the LRN + JOL condition. We found that items with higher JOL (i.e., r minus f) elicited stronger activity in the left DLPFC (MNI: -26, 32, 54, *Z* = 4.17) that extended more anterior to the rostrolateral PFC (RLPFC, MNI: -14, 66, 10, *Z* = 3.67), VMPFC (MNI: 0, 46, -12, *Z* = 4.37), PCC (MNI: 6, -58, 8, *Z* = 3.56), left middle temporal gyrus (MTG) (MNI: -64, -36, -8, *Z* = 3.86), and left superior lateral occipital cortex (sLOC) extending to the angular gyrus (AG) (MNI: 32, -78, 46, *Z* = 4.71) (**Figure 3**). Items with lower JOL did not elicit stronger activation in any region.

**FIGURE 4:**
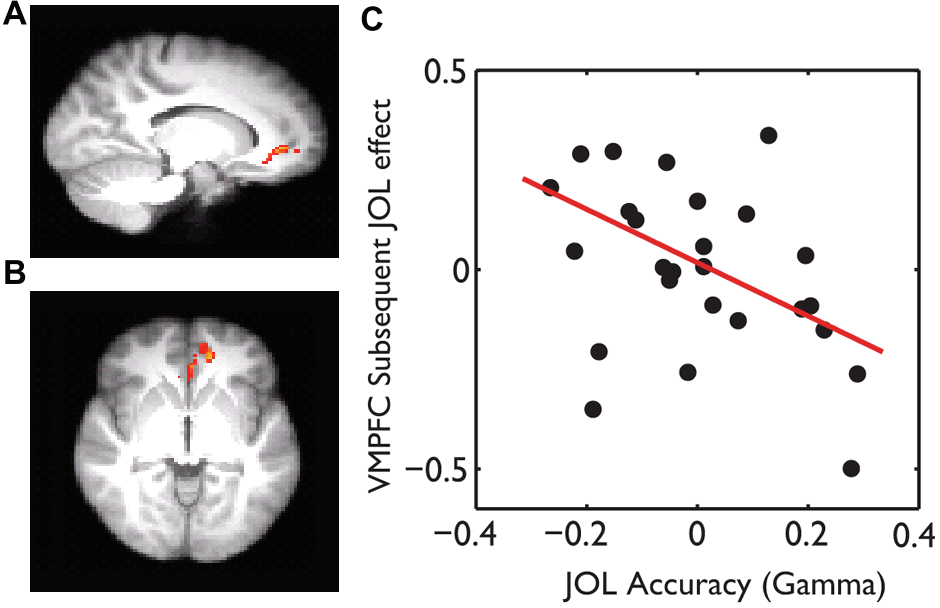
**The effect of JOL accuracy on the subsequent JOL effect.** The VMPFC showed a negative correlation between JOL accuracy (gamma) and the subsequent JOL effect, which was overlain on the saggital **(A)** and axial **(B)** slices of the group average anatomical image. **(C)** Scatter plot of the JOL accuracy and the subsequent JOL effect at the VMPFC. The regression line was based on the robust fit, which was less sensitive to the influence of outliers.

The behavioral data suggest that subjects might use wrong heuristics to guide their JOL and thus had overall low JOL accuracy. If this was the case, subjects who were less affected by the wrong heuristics and thus had higher JOL accuracy would show less subsequent JOL effect in the above regions, including the VMPFC. Consistent with this hypothesis, whole-brain robust regression revealed significant negative correlations between gamma and the subsequent JOL effect in the VMPFC (MNI: -14, 42, -4, *t* = -3.11, small volume corrected using the VMPFC as a mask) (**Figure 4**).

### Dissociated Neural Correlates Underlying Subsequent Memory and JOL

**FIGURE 5:**
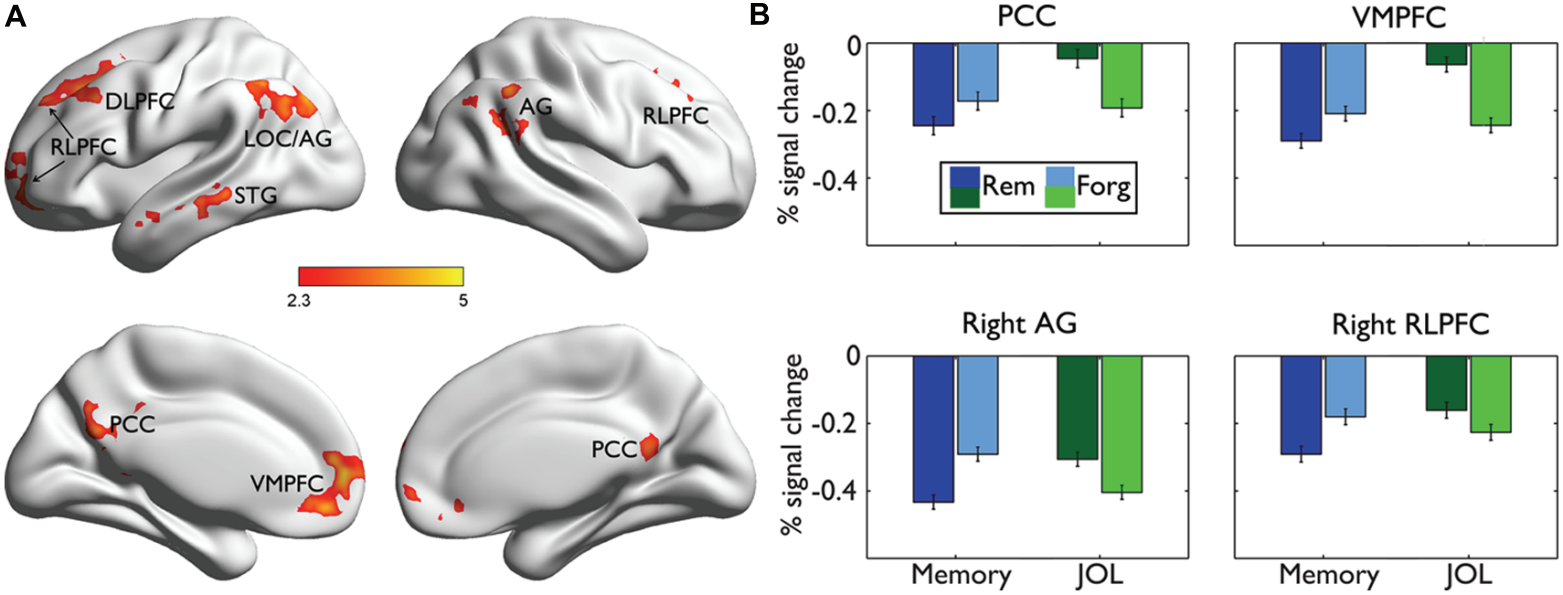
**Comparisons of brain responses underlying subsequent memory performance and JOL. (A)** Brain regions showing significant differences between the subsequent memory effect and the subsequent JOL effect are rendered onto a population-averaged surface atlas (Xia et al., 2013). All activations were thresholded at *Z* > 2.3 (whole-brain corrected *p* < 0.05). **(B)** Bar graphs of the percentage signal change in each ROI, as a function of subsequent memory or JOL. Error bars denote within-subjects standard errors.

Direct comparisons between the neural regions supporting subsequent memory (LRN trials) and subsequent JOL (LRN + JOL trials) revealed significant differences in several regions including the VMPFC (MNI: 0, 48, -8, *Z* = 4.42), PCC (MNI: 0, -66, 30, *Z* = 4.06), left RLPFC (MNI: -26, 40, 40, *Z* = 3.81), right RLPFC (MNI: 26, 30, 46, *Z* = 3.45), left sLOC/AG (MNI: -36, -76, 46, *Z* = 4.11), right AG (MNI: 56, -54, 30, *Z* = 3.72), and right MTG/STG (MNI: -58, -2, -16, *Z* = 3.58) (**Figure 5A**). Further ROI analysis indicated that in four of these regions, including the left VMPFC, PCC, right RLPFC, and right AG, stronger activity was associated with higher JOL, but worse memory performance (**Figure 5B**, **Table 2** for detailed statistics).

**Table 2:** Activation differences related to later memory performance and JOL.

Regions	JOL				Memory performance			
	*r*	*f*	*T*	*p*	*R*	*F*	*T*	*p*
VMPFC	-0.06	-0.24	4.50	0.0001***	-0.29	-0.21	-2.14	0.0425*
PCC	-0.20	-0.19	3.36	0.0025**	-0.25	-0.17	-2.12	0.0363*
L SFG	0.06	-0.12	5.15	< 0.0001****	-0.11	-0.09	-0.78	0.4423
R SFG	-0.16	-0.23	1.83	0.0799	-0.29	-0.18	-3.13	0.0044**
L AG	-0.14	-0.34	4.43	0.0002***	-0.39	-0.34	-1.12	0.2743
R AG	-0.30	-0.40	2.42	0.0231*	-0.43	-0.29	-4.01	0.0005***
L STG	0.02	-0.19	5.69	< 0.0001****	-0.15	-0.10	-1.61	0.1208

*r, will be remembered; *f*, will be forgotten; R, remembered; F, Forgotten. ^∗^*P* < 0.05, ^∗∗^*P* < 0.01, ^∗∗∗^*P* < 0.001, ^∗∗∗∗^*P* < 0.0001.*

### Dissociated Functional Connectivity Underlying Subsequent Memory and JOL

**FIGURE 6:**
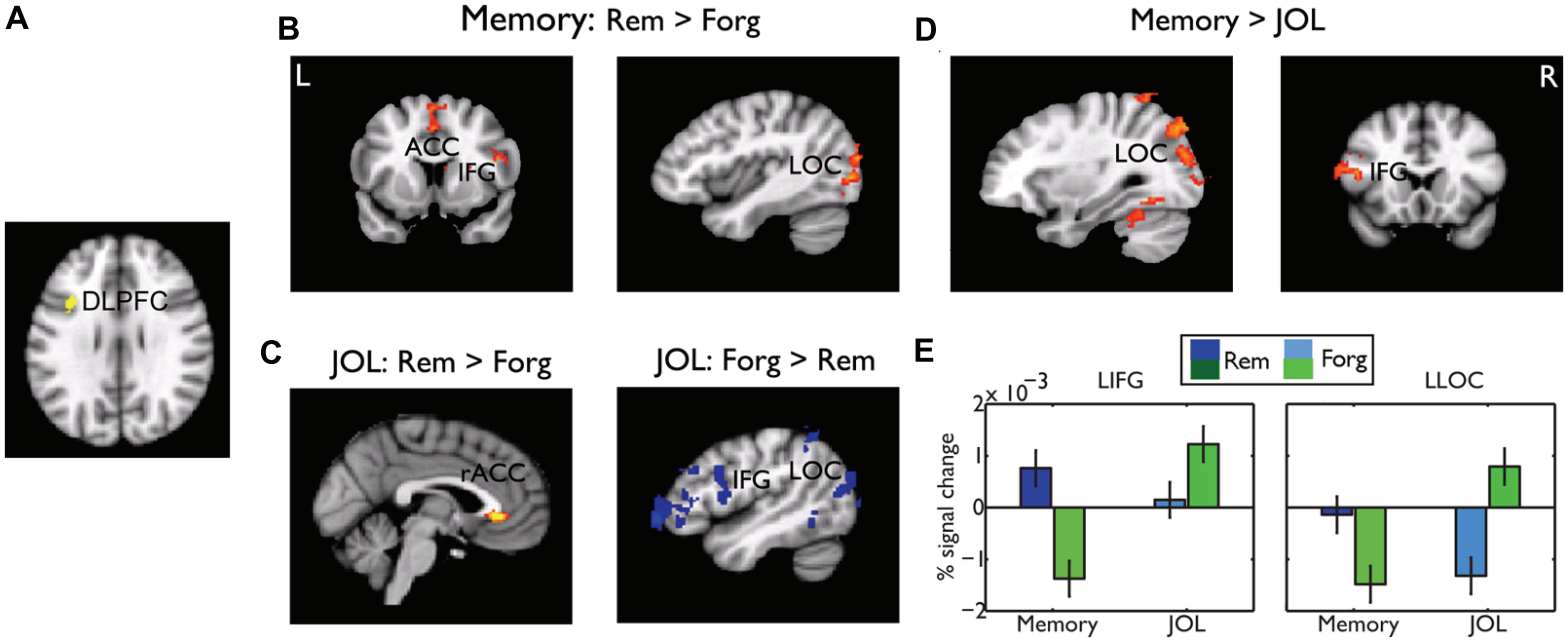
**Psychophysiological interaction (PPI) results. (A)** The seed region for the PPI analysis, the DLPFC, which showed a common effect for memory performance and JOL. **(B)** Brain regions showing stronger functional connectivity with the DLPFC for remembered than forgotten trials. **(C)** Brain regions showing stronger (red-yellow color, uncorrected, *p* < 0.01) and weaker (blue color) functional connectivity for “will be remembered” than “will be forgotten” trials. **(D)** Brain regions showing significant differences in functional connectivity between memory performance and JOL. Unless otherwise stated, all activations were thresholded at *Z* > 2.3 (whole-brain corrected *p* < 0.05). **(E)** Bar graphs of the functional connectivity in the left LOC and IFG, as a function of subsequent memory or JOL. Error bars denote within-subjects standard errors.

Whereas the frontal regions related to memory performance were mostly ventral and those related to JOL were mostly dorsal, conjunction analysis revealed one small cluster in the left DLPFC (centered at -38, 12, 32 in MNI coordinates) that showed a common effect (**Figure 6A**). We did a PPI analysis to examine whether this region played a common or differential role in memory performance and JOL. Using this region as a seed region, we found that subsequently remembered LRN trials, as compared to forgotten LRN trials, were associated with stronger functional connectivity between the left DLPFC and the LOC (MNI: -28, -72, -10, *Z* = 3.92), and between the left DLPFC and the SMA/ACC (MNI: -2, 4, 52, *Z* = 3.4) (**Figure 6B**). In contrast, LRN + JOL trials with higher JOL, as compared to those with lower JOL, were associated with stronger connectivity with the rostral ACC (MNI: -2, 34, -2, *Z* = 3.35, uncorrected) and weaker connectivity with the left occipital cortex (MNI: -28, -90, -0, *Z* = 3.22) and the left IFG/OFG (MNI: -50,40,0, *Z* = 3.98) (**Figure 6C**). Direct comparisons revealed significant differences in the left IFG (MNI: -54, 14, 20, *Z* = 4.04) and the left LOC (MNI: -28, -90, -0, *Z* = 4.85) (**Figures 6D,E**).

## Discussion

One important question in metamemory is why sometimes memory predictions and actual memory performances are dissociated. In the current study, we used an immediate JOL paradigm that has been shown to be able to maximize the dissociation between actual memory performance and JOL (Nelson and Dunlosky, 1991). We found that despite their moderate level of memory performance, participants’ metamemory (or JOL accuracy) was barely at the chance level. With functional imaging and an improved design that could clearly separate the encoding and the JOL processes, we found dissociated neural correlates that supported JOL and subsequent memory performance. These results should deepen our understanding of the cognitive and neural mechanisms of metamemory, and thus help to answer the question of how this dissociation occurs.

The most important finding was that there were dissociated neural processes underlying JOL and later memory performance. Although previous studies already reported dissociations of neural processes during encoding that supported later memory accuracy and retrospective judgment of memory strength (Qin et al., 2011) or confidence (Chua et al., 2004), the dissociation between later memory and prospective judgment of memory strength found in the present study was more striking: Several of these regions showed exactly the opposite patterns of association with later memory performance and JOL. Specifically, higher JOL was associated with stronger activation in the VMPFC, PCC, right angular gyrus, and RLPFC (Schooler et al., 2011). In contrast, better memory performance was associated with stronger activity in the frontal lobe, posterior material-specific regions, and also stronger deactivation in the VMPFC and PCC (Wagner et al., 1998; Daselaar et al., 2004; Kim, 2011).

The left IFG has been consistently implicated in memory encoding (Wagner et al., 1998; Daselaar et al., 2004; Kim, 2011). The left IFG’s activity increases monotonically with subsequent memory longevity (Liu et al., 2014). Increasing the encoding-related activities, by using deeper encoding (Otten et al., 2001), low word frequency (Chee et al., 2003), spaced repetitions (Xue et al., 2010b; Zhao et al., 2015), and anodal transcranial direct current stimulation (Lu et al., 2015), also enhanced subsequent memory. As the left IFG is not involved in memory storage *per se*, it has been implicated in goal-directed task processing that could enhance the cortical representation of item-specific features during encoding (Xue et al., 2013), thus facilitates input to the MTL (medial temporal lobe) where long-term memory is formed.

In contrast, the VMPFC and PCC are two key parts of the default-mode network (DMN; Buckner et al., 2008; Biswal et al., 2010), which is activated when attention is directed internally to self-referential thoughts (Northoff et al., 2006; Buckner and Carroll, 2007). A strong DMN activity often suggests a disengagement of attention to the current task, and is often associated with worse task performance (Buckner et al., 2008; Schooler et al., 2011). Consistent with this view, memory studies have shown that DMN deactivation during encoding was related to better memory performance (Duverne et al., 2009; Uncapher and Wagner, 2009; Kim, 2011; Huijbers et al., 2012).

This dissociation was further supported by functional connectivity results. Only a small cluster in the left DLPFC predicted both memory performance and JOL, but it showed strikingly opposite functional connectivity with the visual cortex and the left IFG that underlain subsequent memory performance and subsequent JOL. In particular, we found that strong functional connectivities between the DLPFC and left IFG and between the DLPFC and the left LOC were associated with better subsequent memory performance but lower JOL score. The connectivity between PFC and occipital cortex has been implicated in effective memory encoding (Vidal-Piñeiro et al., 2014). Excitatory TMS over the PFC could enhance its functional connectivity with the occipital lobe and this increase was positively correlated with TMS effect on memory performance (Vidal-Piñeiro et al., 2014).

Our result could help to clarify the role of the DLPFC in memory encoding and JOL. The DLFPC was also found to be associated with JOL in a previous study (Kao et al., 2005). In that study, JOL and encoding were not separated, thus its result was subject to two alternative interpretations. One possibility is that increased DLPFC activity signals increased effort at encoding, which influences both memory performance and JOL. The other explanation is that increased DLPFC activity reflected partial retrieval of the target in working memory. Neither of the two explanations seems consistent with the current finding. First, because the DLPFC activation was found during the encoding stage, it should not reflect the retrieval of the target in working memory. Second, this region is more dorsal to the regions involved in semantic elaboration, including the IFG and OFC. Third, the reduced functional connectivity for high later JOL, together with enhanced deactivation in the default mode network, suggested that weaker processing led to high JOL, but not the opposite as suggested by previous studies.

Existing cognitive findings suggest that JOL is based on processing fluency whereas actual memory requires desirable difficulties (see Introduction). In these studies, processing fluency or desirable difficulties were measured by reaction time. That is, longer processing time is associated with lower JOL but actually better memory. Using fMRI that could measure the strength of neural activity and functional connectivity, we found that the pattern (i.e., strong DMN activation for high JOL) parallels with the behavioral observations, although we did not find any differences in reaction time or accuracy. In addition, we also found that the neural activity in the PFC was a more reliable predictor of subsequent memory performance than was RT (Liu et al., 2014). In existing behavioral studies, processing fluency is often defined as the subjective experience of ease or difficulty associated with a mental process, which has been operationalized as processing speed. This approach, however, has been criticized for being unable to account for the entire range of fluency effects (Oppenheimer, 2008). Furthermore, manipulations that increased the subjective disfluency did not change the reaction time (Tourangeau and Ellsworth, 1979). Presumably, the neural activity, including that in the DMN and PFC, could provide a more sensitive measure of processing fluency. Future studies need to link the behavioral and neural responses with the subjective feeling, which could significantly deepen our understanding of the mechanisms of JOL.

The low JOL accuracy enabled us to dissociate the neural substrates of subsequent memory and JOL. For several reasons, we did not think the overall chance level of the JOL accuracy would affect the significance of the subsequent JOL effect. First, unlike the subsequent memory effect where a chance level performance would unlikely provide meaningful insights into the neural mechanisms of memory encoding, the JOL is subjective in nature. Therefore, whether they were correct or wrong, JOLs’ neural mechanisms can be studied using the subsequent JOL effect. Second, a chance level performance does not mean that subjects made the decision randomly. Instead, it may be guided by false heuristics. As shown by the behavioral results, subjects gave more low JOL ratings (i.e., 1) to better remembered trials (i.e., scored 4). Importantly, when correlating the JOL accuracy with the subsequent memory effect in the VMPFC, we found that subjects with low JOL accuracy showed a stronger subsequent JOL effect. Together, the behavioral and neural imaging evidence is quite consistent with the heuristic hypothesis (Kelley and Jacoby, 1996; Hertzog et al., 2003; Rhodes and Castel, 2008; Kornell et al., 2011; Sungkhasettee et al., 2011; Besken and Mulligan, 2013).

Our results emphasize the necessity to clearly dissociate the JOL and memory encoding processes. Consistent with the hypothesis that the JOL could serve as an additional encoding process, we found that the LRN + JOL trials were better remembered than the LRN trials. Since subjects often are insensitive to the role of future practice on memory (Koriat et al., 2002; Kornell et al., 2011), this process may have further lowered the JOL accuracy as our subjects were overall under-confident about their memory. More importantly, due to the facilitative role of the JOL in memory encoding, it could contaminate the subsequent memory effect. Consistently, we found that the subsequent memory effect based on the LRN + JOL trials was overall reduced, and no significant deactivations were observed in the PCC and VMPFC. This result could well explain why a previous study did not reveal this dissociation when all learning trials were followed by the JOL (Do Lam et al., 2012).

Several questions need to be further examined. First, the JOL could happen at different stages of learning and may rely on different cues and strategies. For example, delayed JOL is much more accurate than immediate JOL (Nelson and Dunlosky, 1991; Weaver and Kelemen, 1997). Our study provides a useful experimental paradigm for future studies to compare the neural mechanisms underlying different types of JOL. Second, one way to improve the JOL accuracy is to change the decision heuristic by practice and feedback (Koriat and Bjork, 2006), and how that affects the neural mechanisms of JOL deserves further examination. These questions are very interesting as they could reveal whether the same neural processes that support subsequent memory are used to guide JOL, when the JOLs are (moderately) accurate. Third, the current study used familiar words as learning materials and focused on the recognition task. It remains to be examined whether the same pattern is implicated in memory of dynamic, naturalistic and complex materials, given the task and content-specific mechanisms of memory encoding and retrieval (Kwok and Macaluso, 2015). Fourth, emerging studies have implicated the role of neural pattern similarity in subsequent memory (Xue et al., 2010a) and conscious processing (Schurger et al., 2010); it is thus intriguing to see whether these neural states/processes could be “read out” (Williams et al., 2007) to guide meta-cognitions, such as the JOL. Finally, future studies should examine the interaction between the JOL and memory encoding under different LRNs (e.g., self-paced), as previous studies suggested that the JOL plays an important role in guiding self-regulated learning (Thiede, 1999; Metcalfe and Finn, 2008).

## Conclusion

In sum, the present study found a striking dissociation between the neural processes that support later memory performance and later JOL. Strong DMN activities, and weak functional connectivity between frontal and occipital regions, which indicate weak task processing, were associated with high subsequent JOL but low subsequent memory performance. These findings should deepen our understanding of the neural basis of human metamemory bias.

## Author Contributions

HY and GX designed the experiment. HY, YC, QL, XZ performed study. HY, QW, and GX analyzed the data. HY, CC, and GX wrote the manuscript.

## Conflict of Interest Statement

The authors declare that the research was conducted in the absence of any commercial or financial relationships that could be construed as a potential conflict of interest.

## Footnotes

1 www.psychtoolbox.org

2 www.fmrib.ox.ac.uk/fsl
